# The Guizhi Gancao Decoction Attenuates Myocardial Ischemia-Reperfusion Injury by Suppressing Inflammation and Cardiomyocyte Apoptosis

**DOI:** 10.1155/2019/1947465

**Published:** 2019-01-21

**Authors:** Yuan Gao, Ge Song, Ying-Jie Cao, Kui-Po Yan, Bin Li, Xin-Feng Zhu, You-Ping Wang, Zuo-Ying Xing, Lin Cui, Xiao-Xiao Wang, Ming-Jun Zhu

**Affiliations:** ^1^First Affiliated Hospital, Henan University of Traditional Chinese Medicine, Zhengzhou, China; ^2^Henan Province Hospital of Traditional Chinese Medicine, Zhengzhou, China

## Abstract

Guizhi Gancao Decoction (GGD) is a well-known traditional Chinese herbal medicine for the treatment of various cardiovascular diseases, such as myocardial ischemia-reperfusion (I/R) injury and arrhythmia. However, the mechanism by which GGD contributes to the amelioration of cardiac injury remains unclear. The aim of this study was to investigate the potential protective role of GGD against myocardial I/R injury and its possible mechanism. Consistent with the effect of the positive drug (Trimetazidine, TMZ), we subsequently validated that GGD could ameliorate myocardial I/R injury as evidenced by histopathological examination and triphenyltetrazolium chloride (TTC) staining. Moreover, terminal deoxynucleotidyl transferase dUTP nick end labeling (TUNEL) assay demonstrated that GGD suppressed myocardial apoptosis, which may be related to the upregulation of Bcl-2, PPAR*α*, and PPAR**γ** and downregulation of Bax, caspase-3, and caspase-9. Pretreatment with GGD attenuated the levels of proinflammatory cytokines including tumor necrosis factor-*α* (TNF-*α*), interleukin- (IL-) 6, and IL-1*β* in serum by inhibiting Toll-like receptor 4 (TLR4)/NF-*κ*B signaling pathway. These results indicated that GGD exhibits cardioprotective effects on myocardial I/R injury through inhibition of the TLR4/NF-*κ*B signaling pathway, which led to reduced inflammatory response and the subsequent cardiomyocyte apoptosis.

## 1. Introduction

Cardiovascular disease is one of the leading causes of human morbidity and mortality in developed countries [1]. In the United States alone, approximately one million people per year suffer from a myocardial infarction (MI) [2]. Ischemic heart disease is predicted to be the largest threat to human life by 2020 [3, 4]. Currently, the best way of myocardial salvage from ischemia for MI appears to be restoration of blood flow to the ischemic area by thrombolysis, percutaneous transluminal coronary angioplasty, and coronary artery bypass surgery. However reperfusion itself also causes additional damage to the myocardia, called myocardial ischemia/reperfusion (I/R) injury, which results in increased cardiomyocyte death and consequently reduced cardiac function [5]. Therefore, fully understanding the mechanism of I/R injury and seeking for newly effective drugs are still the focuses of intense research.

Inflammatory response and cardiomyocyte apoptosis have been recognized as hallmarks of myocardial I/R injury. Recent evidences suggest that cardiomyocyte apoptosis is primarily triggered or accelerated during I/R and partially contributes to overall cardiomyocyte death [6–8]. Inhibition or interruption of the apoptotic process may prevent the loss of contractile cells, minimize cardiac injury induced by I/R, and delay the occurrence of myocardial stunning and heart failure [9, 10]. Likewise, inflammation plays a key role in the pathophysiology of myocardial I/R injury [11, 12]. Reducing inflammatory responses during reperfusion after ischemic insult has been shown to be beneficial in many studies [13, 14]. In addition, the Toll-like receptor 4 (TLR4)/NF-*κ*B signaling pathway has been recently found to be a potential therapeutic target in the treatment of myocardial I/R injury [15, 16]. Accumulating researches have demonstrated that the TLR4/NF-*κ*B signaling pathway plays a substantial role in mediating inflammatory response and cardiomyocyte apoptosis following myocardial I/R injury [15, 17, 18].

Guizhi Gancao Decoction (GGD), originally described in* Treatise on Febrile Diseases* written by Zhongjing Zhang in Eastern Han Dynasty of ancient China, is a famous classic Traditional Chinese Medicine (TCM) formula consisting of two medicinal herbs, namely,* Ramulus Cinnamomi *(RC) and* Radix Glycyrrhizae *(RG) in the ratio of 2:1(w/w). RC and RG have been used extensively in China to treat inflammatory and ischemic diseases [19–21]. The active components in the two individual herbs have been clearly characterized [22, 23]. Cinnamic acid (in RC), liquiritin (in RG), isoliquiritigenin (in RG), and glycyrrhetinic acid (in RG), which can increase coronary circulation [24] and protect against the development of arrhythmia [25–27], are effective constituents of the RC–RG herb pair for the cardiovascular system. In China, GGD has been widely used for centuries to treat cardiovascular diseases in clinic. Previous data from experimental studies have demonstrated that GGD pretreatment significantly attenuated myocardial I/R injury by inhibiting myocardial oxidative stress and Ca^2+^ overload [28, 29], and this observed cardioprotective effect was associated with decreased cardiac arrhythmia after myocardial I/R injury [30, 31]. The potential effects of GGD on myocardial I/R injury prompted us to investigate whether it is capable of exerting protective effects during myocardial I/R injury by its anti-inflammatory and antiapoptotic properties via regulation of the TLR4/NF-*κ*B signaling pathway and what is the underlying mechanism responsible for its actions.

## 2. Materials and Methods

### 2.1. Drugs and Reagents

GGD extract, provided by the First Affiliated Hospital, Henan University of Traditional Chinese Medicine, consisted of RC and RG in a ratio of 2:1. Trimetazidine (TMZ) was produced by Servier Pharmaceutical Co., Ltd., (Tianjin, China). 2,3,5-triphenyltetrazolium chloride (TTC) was purchased from Sigma Chemical Co. (St. Louis, USA). In situ cell death detection kit was purchased from Roche Molecular Biochemicals (Basel, Switzerland). Tumor necrosis factor-*α* (TNF-*α*), interleukin-6 (IL-6), and interleukin-1*β* (IL-1*β*) ELISA kits were purchased from Nanjing KeyGEN Biotech (Nanjing, China). Antibodies against Bcl-2, Bax, caspase-3, caspase-9, PPAR*α*, PPAR***γ***, TLR4, NF-*κ*B p65, and I*κ*B*α* were purchased from Cell Signaling Technology (Danvers, USA).

### 2.2. Animal Treatment

Male Sprague-Dawley rats (250-300g) were purchased from Animal Experiment Center of China Three Gorges University (Certificate no. SCXK 2017-0012). The animals were kept in rooms maintained at 23±2°C in a 12 h light/dark cycle and were fed a standard rodent diet with free access to water following international recommendations. All animal experiments in this study were performed in accordance with China Academy of Chinese Medical Sciences Guide for Laboratory Animals that conforms to the Guide for the Care and Use of Laboratory Animals published by the US National Institutes of Health (NIH Publications number 85-23, revised in 1996). Rats were randomly divided into five groups (n = 18 per group) and treated as follows: (1) the control group; (2) the I/R group; (3) the I/R group treated with GGD solution at doses of 1.8g/kg and 3.6g/kg, respectively; (4) the I/R group treated with TMZ solution at the dose of 10mg/kg. GGD or TMZ was given intragastrically once a day for 14 consecutive days, while the control and I/R groups were given normal saline. The GGD decoction (90g) consisted of RC 60g and RG 30g (according to* Treatise on Febrile Diseases*). The experimental dose of rats was 6.3 times that of human beings, and from that the dose was calculated as RC:6.3*∗*(60*∗*1000/70) =5400mg =5.4 g/kg and RG:6.3*∗*(30*∗*1000/70) =2700mg =2.7 g/kg for rats. But this dose was actually too higher for rats. Referring to other classic prescriptions based on RC–RG herb pair, such as Guiganlongmu Decoction (RC:15g and RG:30g) and Guizhi Decoction (RC:45g and RG:24g), the final dose which was determined was 1.8~3.6g/kg, which was acceptable for gavage in rats.

One hour after the last treatment, the rats were anesthetized with 10% chloral hydrate (300mg/kg, intraperitoneal injection). After tracheal intubation, the rats were ventilated with room air using an animal respirator. Left thoracotomy was performed at the fourth intercostals space and the heart was exposed. By a surgical needle, a 6-0 silk ligature was placed under the left anterior descending coronary artery (LAD) and encircled with a suture. The LAD was ligated to induce ischemia for 30 min. R wave amplification and ST segment depression were observed immediately in lead II of the attached electrode. Myocardial ischemia was indicated if the myocardia distal to the ligation line darkened. After 30 min of ischemia, the slipknot was released and the animal received 120 min of reperfusion. The control group (n=18) was sham-operated animals undergoing the same surgical procedure, except for the fact that the silk ligature placed around the left coronary artery was not tied. Except for accidental deaths due to anesthesia or failed surgery, the numbers of surviving rats in each group were as follows: I/R (n=15), I/R+GGD 1.8g/kg (n=14), I/R+GGD 3.6g/kg (n=12), and I/R+TMZ (n=14). Blood was collected after reperfusion by cardiac puncture and centrifuged at 2000* g *for 10 min. Serum and plasma were stored at -80°C for further analysis. Hearts were removed and rinsed with ice-cold phosphate buffered saline. Ventricular tissue was immediately frozen in liquid nitrogen and stored at -80°C.

### 2.3. Assessment of Myocardial Infarct Size

The rats were sacrificed at 120min of reperfusion, and their hearts were immediately removed and frozen at -20°C for 15 min and then cut into five cross-section slices. Heart slices were incubated for 15 min in 1% TTC at 37°C. TTC-stained areas (red staining, ischemic areas) and non-TTC-stained areas (white, infarct areas) were determined. The infarct size was analyzed by Image-Pro Plus image analysis software. Proportions of infarct myocardium to the whole myocardium tissues were then calculated using the following formula: Infarct area/whole heart area (INF/WH%).

### 2.4. Hematoxylin–Eosin (HE) Staining and Immunohistochemical Evaluation

Myocardial tissues were rapidly transferred to prepare 4% formaldehyde (Sangon Biotech, China) and fixed overnight at 4°C before they were gradiently dehydrated with ethanol. The tissues were then embedded in paraffin, cut into 5*μ*m-thick sections, and evaluated for morphological changes with HE staining under a light microscope at a magnification of ×200. For immunohistochemical analyses of TLR4, tissue sections were deparaffinized and blocked with 10% rabbit serum to prevent nonspecific antibody binding. After washing three times with phosphate buffer saline (PBS), which contained (in g/L) NaCl 8, KCl 0.2, Na_2_HPO_4_ 3.48, and KH_2_PO_4_ 0.2, the sections were then incubated with TLR4 primary antibody at 4°C overnight. The sections were washed three times with PBS for 5min and incubated with secondary antibody at 37°C for 1 h. The sections were rinsed again with PBS for three times, incubated with diaminobenzidine for 10-15min, counterstained with hematoxylin, dehydrated, and mounted for microscopic observations with a magnification of ×400.

### 2.5. Terminal Transferase-Mediated dUTP Nick End Labeling Staining (TUNEL)

Myocardial apoptosis was detected by TUNEL staining using in situ cell death detection kit. TUNEL assay was performed according to the manufacturer's protocol. Brown colored positive apoptotic cells were observed with TUNEL method and visualized at ×400 magnification.

### 2.6. Protein Extraction and Western Blotting Analysis

Protein was extracted from myocardial tissues for Western blotting analysis. Briefly, myocardial tissues were homogenized with a glass dounce homogenizer in RIPA buffer (50 mM Tris-HCl, pH 7.5, 150 mM NaCl, 2 mM EDTA, 1% NP40, 1% Triton X-100, 0.1% SDS, and 0.5% sodium deoxycholate) and then centrifuged at 20,000×*g* for 15 min. The supernatants were collected for Western blotting and the protein concentrations were determined by Bradford assay (Bio-Rad, Hercules, CA, USA). Equal amounts of proteins were separated by 12 % sodium dodecyl sulfate polyacrylamide gel electrophoresis (SDS-PAGE) and transferred to polyvinylidene fluoride (PVDF) membranes (Bio-Rad, Hercules, CA, USA). Membranes were blocked with 5 % nonfat dry milk and then washed. Primary antibodies, including antibodies against Bcl-2 (1:1000), Bax (1:1000), caspase-3 (1:1000), caspase-9 (1:1000), PPAR*α* (1:1000), PPAR**γ** (1:1000), TLR4 (1:1000), NF-*κ*B p65 (1:1000), and I*κ*B*α* (1:1000), were used to incubate the membranes overnight at 4°C. Horseradish peroxidase-conjugated secondary antibody (Cell Signaling Technology, Danvers, USA) was used to incubate the membrane for 2h. Immunoreactivity was detected by ECL reagents (Nanjing KeyGEN Biotechnology, China) and a gel imaging system (Tanon Science & Technology Co., Ltd., China) was used to visualize the protein bands.

### 2.7. Enzyme-Linked Immunosorbent Assay (ELISA)

The serum levels of TNF-*α*, IL-6, and IL-1*β* were analyzed spectrophotometrically according to the instruction of ELISA kits.

### 2.8. Statistical Analysis

All statistical analyses were performed using SPSS 16.0 software. Data were expressed as mean ± standard error (SEM) and analyzed using one-way ANOVA followed by Tukey's post hoc test for multiple comparisons. *P* values less than 0.05 were considered statistically significant.

## 3. Results

### 3.1. Effect of GGD on Infarct Size

**Figure 1:**
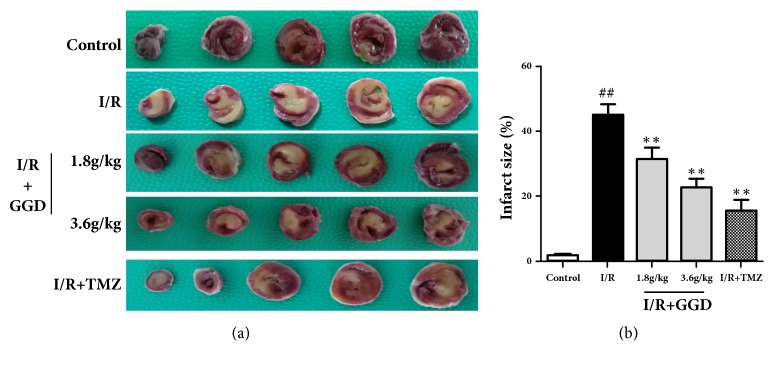
Effect of GGD treatment on infarct size (INF/WH %) in each group. (a) Representative TTC staining of samples from rat ventricles subjected to different treatments. (b) Quantitative densitometric analysis of myocardial infarct sizes (INF/WH%). Values were presented as mean±SEM. n=6; ^#^*P*<0.05 and^ ##^*P*<0.01 compared with control group; *∗P*<0.05 and *∗∗P*<0.01 compared with I/R group.

Myocardial infarct size was assessed in the current study by TTC staining. As shown in Figure 1, the white color represents the infarct area, and the red color the normal myocardial tissue. Compared with the I/R group, treatment with TMZ and GGD at doses of 1.8g/kg and 3.6g/kg significantly reduced the sizes of myocardial infarction.

### 3.2. Effect of GGD on Myocardial Morphological Changes

**Figure 2:**
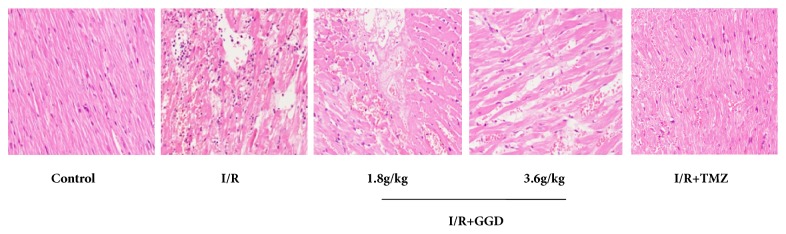
Effect of GGD treatment on histopathological changes in myocardial tissues of different groups. Control group showed obvious integrity of myocardial membrane without any inflammatory infiltration, cardiac necrosis, or infarction. I/R group showed distorted cardiac muscles, local swelling, inflammatory infiltration, and cardiac necrosis. Treatment with TMZ and GGD at doses of 1.8g/kg and 3.6g/kg reduced inflammatory infiltration and cardiac necrosis. Photomicrographs were taken at ×200 magnification.

In the present study, morphological changes of myocardial tissue were observed by HE staining. As shown in Figure 2, myocardia from the control group were normal in morphology, with intact myocardial membrane and no signs of inflammatory infiltration, cardiac necrosis, infarction, or other pathological changes. In contrast to the control group, I/R group showed distorted cardiac muscles, local swelling, inflammatory infiltration, and cardiac necrosis. However, treatment with TMZ and GGD at doses of 1.8g/kg and 3.6g/kg exerted obvious ameliorative effect on the above morphological changes by reducing inflammatory infiltration and cardiac necrosis.

### 3.3. Effect of GGD on Cardiomyocyte Apoptosis

**Figure 3:**
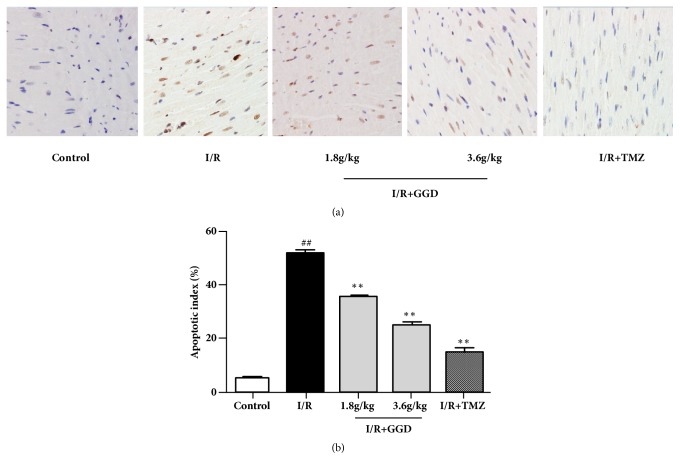
Effect of GGD treatment on cardiomyocyte apoptosis in each group as detected by TUNEL assay. (a) Representative TUNEL staining of samples from rat ventricles subjected to different treatments. Apoptotic cardiomyocytes were stained brown, whereas TUNEL-negative cells were stained blue. Photomicrographs were taken at ×400 magnification. (b) Percentages of apoptotic cardiomyocytes. Values were presented as mean±SEM. n=6; ^#^*P*<0.05 and^ ##^*P*<0.01 compared with control group; *∗P*<0.05 and *∗∗P*<0.01 compared with I/R group.

TUNEL assay was employed to ascertain the antiapoptotic effect of GGD. Apoptosis was determined by using a TUNEL detection kit according to the manufacturer's instructions. As shown in Figure 3, TUNEL-positive cells were considered apoptotic cells, as represented by their brown color. Myocardial apoptosis of I/R group was significantly increased compared to the control group. However, treatment with TMZ or GGD at doses of 1.8g/kg and 3.6g/kg markedly reduced myocardial apoptosis.

### 3.4. Effect of GGD on Expression of Apoptosis-Related Proteins

**Figure 4:**
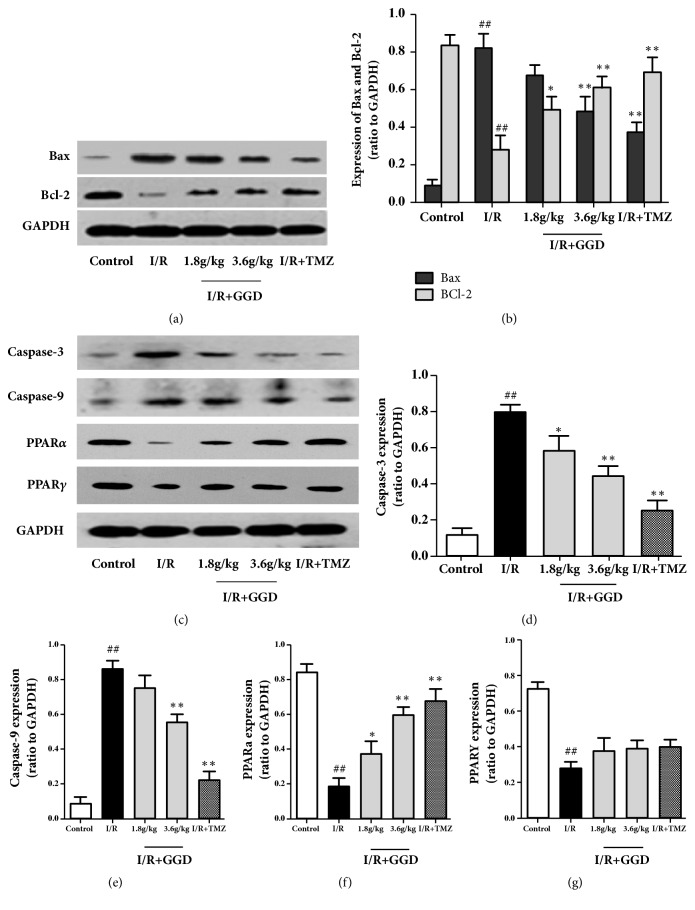
Effect of GGD treatment on expressions of apoptosis-related proteins in each group as detected by Western blot. (a,c) Representative immunoblots of samples from rat ventricles subjected to different treatments. (b, d–g) Quantitative densitometric analysis of Bax, Bcl-2, caspase-3, caspase-9, PPAR*α*, and PPAR**γ**, with GAPDH as an internal control. Values were presented as mean±SEM. n=4; ^#^*P*<0.05 and^ ##^*P*<0.01 compared with control group; *∗P*<0.05 and *∗∗P*<0.01 compared with I/R group.

To further demonstrate the antiapoptotic mechanism of GGD, we evaluated the expressions of apoptosis-related proteins by Western blotting analysis (Figure 4). Results of our current study showed that myocardial Bax protein expression was significantly increased, while the Bcl-2 expression was decreased in the I/R group as compared with the control group (Figures 4(a) and 4(b)). As with the positive drug TMZ, GGD administration increased the expression of Bcl-2 at doses of 1.8g/kg and 3.6g/kg but decreased the expression of Bax at the dose of 3.6g/kg, as compared with those in myocardia from the I/R group. Compared with the control group (Figures 4(c)–4(g)), the expression levels of caspase-3 and caspase-9 were upregulated by I/R injury, whereas the protein expressions of PPAR*α* and PPAR*γ* were downregulated. To different extents, treatment with TMZ or GGD reversed the changes in apoptosis-related protein expressions induced by I/R. Among them, PPAR*γ* expression was upregulated, but not significantly as compared with the IR group.

### 3.5. GGD Reduced TLR4 Expression in Myocardia Subjected to I/R

**Figure 5:**
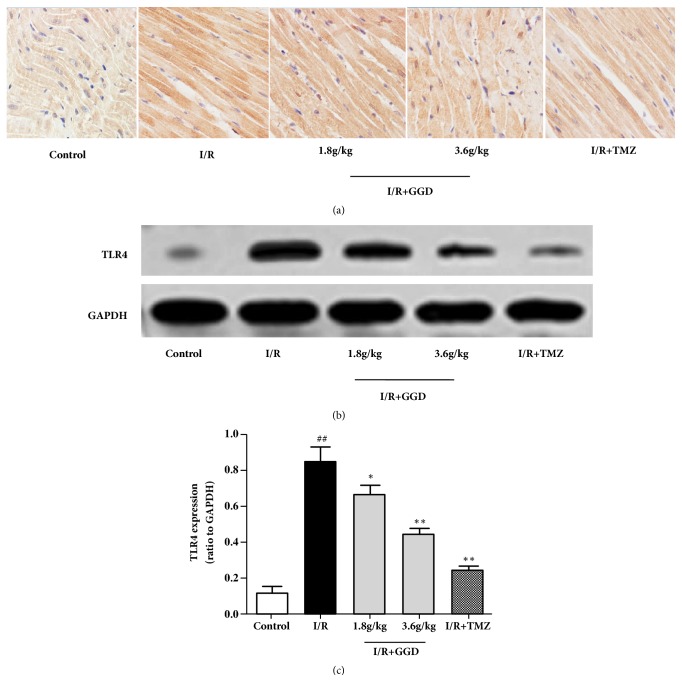
Effect of GGD treatment on myocardial TLR4 expression in each group. (a) Representative immunohistochemistry of TLR4 from rat ventricles. The brown color in the cytoplasm represents the expression of TLR4. Photomicrographs were taken at ×400. (b) Representative immunoblots of samples from rat ventricles subjected to different treatments. (c) Quantitative densitometric analysis of TLR4 protein with GAPDH as an internal control. Values were presented as mean±SEM. n=4; ^#^*P*<0.05 and^ ##^*P*<0.01 compared with control group; *∗P*<0.05 and *∗∗P*<0.01 compared with I/R group.

Protein level of TLR4 was determined by immunohistochemistry and Western blotting in the current study. As shown in Figure 5, TLR4 expression was significantly increased in the myocardia of I/R group as compared with that from the control group. Interestingly, treatments or TMZ and GGD at doses of 1.8g/kg and 3.6g/kg reduced the protein level of TLR4 in the myocardia as proved by both immunohistochemistry and Western blot, which suggested that inhibition of TLR4 might be involved in the protective effects of GGD on I/R injury.

### 3.6. GGD Reduced NF-*κ*B p65 in Myocardia Subjected to I/R

**Figure 6:**
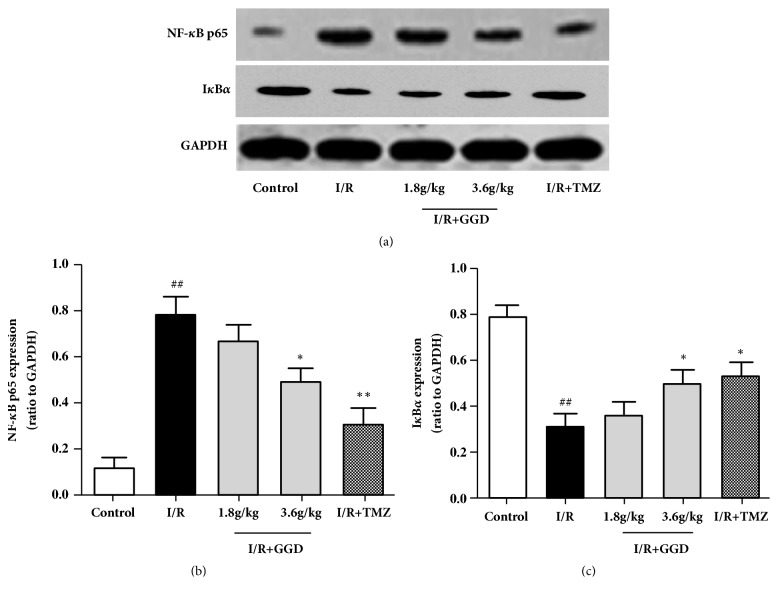
Effect of GGD treatment on myocardial NF-*κ*B p65 and I*κ*B*α* expressions in each group. (a) Representative immunoblots of samples from rat ventricles subjected to different treatments. (b, c) Quantitative densitometric analysis of NF-*κ*B p65 and I*κ*B*α* with GAPDH as an internal control. Values were presented as mean±SEM. n=4; ^#^*P*<0.05 and^ ##^*P*<0.01 compared with control group; *∗P*<0.05 and *∗∗P*<0.01 compared with I/R group.

To further investigate the effect of GGD on the TLR4-mediated signaling pathway, the expression level of NF-*κ*B p65 and its inhibitory protein IKB*ɑ* were determined using Western blotting. The results from Western blotting analyses showed that I/R injury induced an upregulation of NF-*κ*B p65 (Figures 6(a) and 6(b)). In contrast, IKB*α*, an inhibitor of the NF-*κ*B signaling pathway, was downregulated by I/R injury (Figures 6(a) and 6(c)). The upregulation of NF-*κ*B p65 and downregulation of IKB-*α* were partially abolished by the administration of TMZ or GGD at the dose of 3.6g/kg.

### 3.7. GGD Inhibited Proinflammatory Cytokines in the Serum following I/R Injury

**Figure 7:**
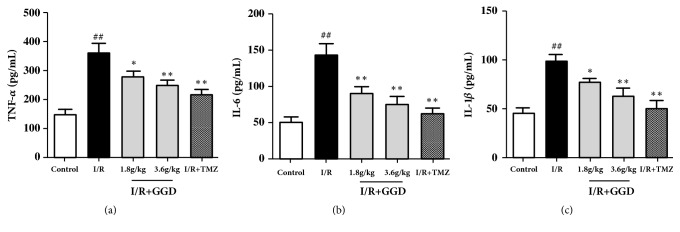
Effect of GGD treatment on proinflammatory cytokine production in each group. GGD treatment significantly reduced the serum levels of TNF-*α* (a), IL-6 (b), and IL-1*β* (c) following I/R injury. Values were presented as mean±SEM. n=6; ^#^*P*<0.05 and^ ##^*P*<0.01 compared with control group; *∗P*<0.05 and *∗∗P*<0.01 compared with I/R group.

In order to further verify the effect of GGD on the TLR4/NF-*κ*B signaling, the serum levels of proinflammatory cytokines, including TNF-*α*, IL-6, and IL-1*β*, were determined in rats from each group. As shown in Figure 7, treatment with TMZ or GGD at doses of 1.8g/kg and 3.6g/kg significantly reduced the serum levels of TNF-*α*, IL-6, and IL-1*β* as compared with the I/R group, demonstrating that treatment with GGD might confer its cardioprotective effect by reducing proinflammatory cytokines in I/R injury.

## 4. Discussion

There is an unmet need for developing an innovative cardioprotective modality for acute MI, where interventional reperfusion therapy is hampered by IR injury. Even though accumulating evidence suggested that statin therapy could ameliorate myocardial I/R injury and improve myocardial and coronary functions after cardiac surgery, the effect of statins on myocardial I/R injury in cardiac surgery is controversial [32, 33]. Chinese herbal medicines have long been used as alternative therapy against myocardial I/R injury because of their anti-inflammatory, antioxidative, antiapoptotic effects. GGD, first descripted in* Treatise on Febrile Diseases*, is mainly used to treat cardiovascular diseases, such as myocardial IR injury and arrhythmia. However, the mechanisms remain poorly known. The present study examined, for the first time, the cardioprotective effects of GGD, which worked by attenuating myocardial infarction size and improving morphological disorders in myocardial issues of rats subjected to I/R injury. These potential effects of GGD may be associated with the suppression of TLR4/NF-*κ*B signaling pathway through inhibition of serum inflammatory cytokines and reduction of cardiomyocyte apoptosis induced by I/R injury. It was implied that pretreatment with GGD probably elicited anti-inflammatory and antiapoptotic effects against I/R via modulation of the TLR4/NF-*κ*B signaling pathway.

It is well known that reperfusion is the foundation of therapy to protect the ischemic myocardia from further damage. Paradoxically, restoration of blood flow initiates an intense local and systemic inflammatory response that may aggravate myocardial injury and adversely affect the recovery of left ventricular function after ischemia [34]. Pattern recognition receptor TLR4 participates in both immune response and inflammatory response following I/R injury [35, 36]. Previous research suggested that the increased expression of TLR4 played a vital role in the significant inflammatory response to the I/R injury [37]. Further study showed that TLR4-deficient mice (C3H/HeJ) had smaller infarct size and exhibited less severe inflammation compared with control mice after I/R injury [38]. This is consistent with the belief that besides its role in innate immune responses, TLR4 serves as a proinflammatory agent in murine myocardial I/R injury. Also, studies have exhibited that the effect of TLR4- mediated inflammatory response in I/R injury was related to activation of NF-*κ*B and other proinflammatory cytokines including TNF-*α* and IL-1*β* [39, 40]. RC and RG have been shown to effectively inhibit proinflammatory cytokine production because of their immunomodulatory effects [19–21]. In our study, administration of GGD before I/R injury significantly decreased the expressions of TLR4 and NF-*κ*B. This was accompanied by decreased inflammatory response as shown by lowered TNF-*α*, IL-6 and IL-1*β* levels, which may underlie the effects of reducing infarct area and improving morphological changes. These results demonstrated that GGD may confer a cardioprotective effect to I/R injury probably due to its anti-inflammatory properties.

Cardiomyocyte apoptosis proves to be a major contributor to myocardial reperfusion injury, which is primarily triggered by ischemia and aggravated during the process of reperfusion and partially results in overall cardiomyocyte death [41]. Inhibition of myocardial apoptosis protects the myocardial tissues against I/R injury and improves the cardiac function [42]. Furthermore, the development of myocardial apoptosis has been demonstrated to be associated with the TLR4-related pathway and inflammatory cytokines such as TNF-*α* and IL-6 [43]. Cell apoptosis is induced by the accumulating TNF-*α* after reperfusion interaction with TNF-*α* receptor type 1, which in turn activates further inflammatory response [44–46]. Subsequently, the amplified inflammatory response and apoptosis had joint effect in causing deterioration of the cardiac function. We used two methods to detect myocardial apoptosis in order to further investigate the protective mechanism of GGD against I/R injury. The TUNEL assay showed that I/R injury induced a higher apoptotic rate in I/R group compared to control group. Pretreatment with GGD resulted in remarkable reduction in the percentage of apoptotic cardiomyocytes. As an important mitochondrial regulator during myocardial apoptosis, Bcl-2 exerts an antiapoptotic effect by blocking the release of cytochrome c and downregulating caspase activity [47]. Apoptosis-related proteins, such as Bax, caspase-3, and caspase-9, also play pivotal roles in apoptosis [48]. The caspase-independent apoptotic pathway responds to death signals by releasing apoptosis-inducing factors from the mitochondrial intermembrane space, which are then translocated to the nucleus [49]. PPARs have a protective effect on myocardia during I/R injury development because it can regulate the gene expressions related to the lipid and energy metabolism and inflammatory response [50]. Ligands of PPAR*γ* and PPAR*α* have been demonstrated to reduce myocardial infarct size in I/R; therefore, PPAR agonists may be useful in conditions associated with I/R injury of the heart and other organs [51–53]. Activation of PPAR*γ* has pleiotropic effects in vasculature, including anti-inflammatory, antioxidative, antiapoptotic, and anti- hypertensive effects [54, 55]. The present study revealed that pretreatment with GGD significantly reduced the expressions of Bax, Caspase-3 and Caspase-9 proteins, while the expressions of Bcl-2, PPAR*α*, and PPAR**γ** were increased. These results from our current study revealed that both inflammatory cytokines (TNF-*α*, IL-6, and IL-1*β* levels) and apoptotic regulatory proteins were dramatically reduced by pretreatment with GGD as compared with that of the I/R group. These findings were accompanied by decreased infarct size and improved morphological changes, indicating that attenuation of the subsequent myocardial necrosis could be attributed, at least partially, to antiapoptosis at the early stage of I/R.

In conclusion, the present study demonstrated that pretreatment with GGD could protect the myocardia against I/R injury by downregulation of the TLR4/NF-*κ*B signaling pathway and amelioration of subsequent inflammatory response and cardiomyocyte apoptosis.

## Data Availability

The data used to support the findings of this study are available from the corresponding author upon request.
